# Emotional and Cognitive Effects of Simulated Temporary Hearing Deficit with Healthy Adults

**DOI:** 10.3390/audiolres16010013

**Published:** 2026-01-19

**Authors:** Leora Moss Levy, Kinneret Weisler

**Affiliations:** 1 Department of Communication Disorders, Achva Academic College, Arugot 7980400, Israel; kineret00@gmail.com; 2 Kidma Center for Hearing Tests, Hearing Aids, and Speech Therapy, Jerusalem 9446724, Israel

**Keywords:** temporary hearing loss, cerumen impaction, earplug simulation, anxiety, mood, attention, cognitive performance, auditory perception

## Abstract

**Background/Objectives**: Accumulation of cerumen (earwax) in the auditory canal is a common condition, particularly in children and older adults, and often causes temporary hearing loss. While chronic hearing loss is known to affect mood and cognition, little is known about the psychological impact of short-term auditory deprivation. This pilot study aimed to examine the emotional and cognitive effects of simulated temporary hearing loss. **Methods**: Thirty healthy adults (16 females, aged 18–60) participated. Temporary hearing loss was simulated by placing earplugs in both ears for two hours. Participants completed four tests, assessing anxiety, mood, and attention at three time points: before wearing earplugs, during the blocked condition, and after earplug removal. **Results:** Participants showed a significant increase in state anxiety and a decrease in mood during the earplug condition. Interestingly, visual attention performance improved while hearing was obstructed and remained elevated even after earplug removal. **Conclusions:** Short-term simulated hearing loss produces measurable emotional and cognitive changes, including increased anxiety but enhanced visual attention. Clinicians should consider these effects when assessing patients with temporary hearing obstruction, such as those with cerumen impaction. The results carry implications for the broader population wearing earplugs on a temporary basis including musicians, construction employees, and, in general, people working in noisy environments.

## 1. Introduction

Accumulation of cerumen or earwax in the outer ear often leads to blockage of the auditory canal and, as a result, to temporary hearing loss [1]. From a physiological perspective, the production of cerumen is a naturally occurring process that helps maintain a healthy outer ear. The substance cleans, protects, and lubricates the external auditory canal. Although it is a beneficial and often harmless biological process, it can lead to conductive hearing loss when impacted. Cerumen impaction can generate feelings of fullness, itching, otalgia, fluid discharge, odor, or cough, and secondary issues can include psychological difficulties. This was the focus of the present study. We sought to uncover the emotional and cognitive effects of cerumen-induced temporary hearing loss. In our study, cerumen impaction was simulated by the artificial placement of earplugs in the auditory canal of healthy individuals. We tested the emotional state and cognitive function during blockage, as well as before and after the condition.

During normal hearing, cerumen is eliminated by self-cleaning mechanisms that remove it from the ear canal [2,3]. This process is prevented by cerumen impaction. Among the adverse consequences of cerumen impaction are problems of diagnosis—complete examination of the external auditory canal and the tympanic membrane becomes difficult, and audiometry and tympanometry are challenging under this condition [2,3]. The condition is more disturbing when the blockage is bilateral, often leading to agitation or ear infections. Despite these symptoms, treatment is simple and provides rapid relief [4].

Cerumen impaction is fairly common, affecting approximately 10% of children, 5% of adults, and over 33% of the elderly population [5]. The rate is similar among children with developmental disorders [6]. In the United States alone, around 12 million people seek help annually for cerumen-related conditions [2]. The problems are exacerbated in the elderly, making diagnosis and communication more difficult [7,8,9].

While chronic hearing loss has been widely studied and linked to poor communication, social isolation, anxiety, and depression [10,11,12,13,14,15], much less is known about the emotional and cognitive effects of temporary hearing loss. Indeed, a recent study [16] noted that “reviews revealed no study of the effect of these devices [earplugs] on emotional states of workers” (p. 22; paratheses provided). The few previous studies found inconsistent results: some reported no significant changes in cognitive performance after cerumen removal [17], whereas others observed improvement in emotional state [18] or mental changes following transient hearing loss [19].

We should note that temporary hearing deficiency is quite widespread in the general population, particularly in specific occupations. A good test case is the temporary deficiency of hearing reported by musicians who wear earplugs. Although inserting plugs is highly recommended to preserve good hearing, the emotional-cognitive effects widely vary. The psychological discomfort can be quite severe so that Chesky et al. [20] conclude that “earplugs… should be considered as a last resort” [p.1].

We should emphasize that, usually, cerumen impaction does not entail total hearing loss of temporary deafness. This situation involves a noticeable drop in the loudness of incoming sounds but, more often than not, speech is still discernible if with effort on the part of the listener. We simulated this situation in the study. We did produce an appreciable attenuation of incoming sounds but the attention still allowed audibility if by a notch.

Given the variable reports in the field and the limited research on cerumen-induced temporary hearing loss in the lab, we sought to examine whether a short-term hearing obstruction can influence mood and cognitive function. Therefore, in the present study, we simulated temporary hearing loss by placing earplugs in both ears of healthy participants. We tested their emotional and cognitive performance before, during, and after the simulated hearing loss condition.

## 2. Materials and Methods

### 2.1. Participants

Thirty healthy participants (16 females), aged 18–60 years (M = 38, SD = 15), were recruited from the Achva Academic College community. All participants reported normal hearing and no known history of auditory or neurological disorders. The study was approved by the Achva College Ethics Committee, and all participants provided written informed consent prior to participation.

### 2.2. Apparatus and Stimuli

Commercial Mack’s^®^ ultrasoft foam earplugs (Plymouth, MI, USA) were used to simulate temporary hearing loss. According to ANSI Standard S3.19-1974, these earplugs provide substantial sound attenuation, averaging at least 37 dB across frequencies and approximately 41 dB at 1000 Hz.

Two tests of emotional state were administered.

(1) The State-Trait Personality Inventory (STPI) by Spielberger et al. (1995) [14], validated in Hebrew [21], assessed state anxiety using 10 items rated from 1 (“not at all”) to 5 (“to a great extent”). The total score ranged from 10 to 50, with higher scores indicating greater anxiety. Reliability in this study was α = 0.762.
(2) The Visual Analog Mood Scale (VAMS) [3,4] consisted of five facial expressions ranging from sad (1) to happy [22,23,24]. The correlation between VAMS and depression scales was r = 0.79 [5].

Two cognitive tests were also used.

(1) The d2 Test of Attention [25] assessed selective attention by requiring participants to identify the letter “d” marked with two short lines. Performance was measured by the total number of correct responses within 20 s per sequence.
(2) The Coding-Digit Subtest from the Wechsler Intelligence Scale [26] measured visual processing speed and accuracy by having participants fill symbols corresponding to digits within two minutes.

### 2.3. Design and Procedure

Testing was conducted individually at participants’ homes over a nine-day period. Let us reiterate that we tested the impact of temporary hearing loss via simulations in adults with healthy ears.

Session 1 (Baseline): Emotional and cognitive tests were administered under normal hearing conditions.

Session 2 (Earplug condition): Approximately one week later, participants wore earplugs in both ears for two hours while performing their regular daily activities, after which the same tests were administered. Prior to donning the earplugs, guidance was given remotely to each participant on how to insert the earplugs. All questions (there were few) were answered on the spot. All participants reported noticeable reduction in loudness. Session 3 (Post condition): The following day, tests were repeated once more without earplugs.

The order of test administration was randomized across participants and across sections with each participant. All participants provided written informed consent, and all data are available upon reasonable request from the corresponding author.

## 3. Results

### 3.1. Anxiety (STPI)

**Figure 1:**
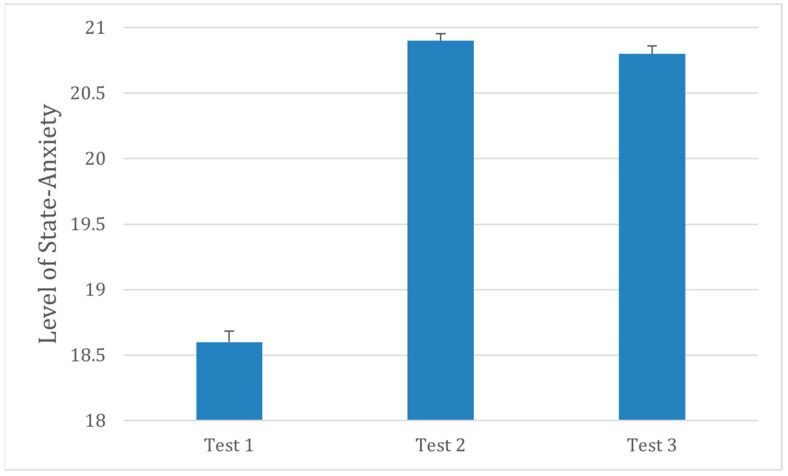
Mean state-anxiety [STPI} scores across testing sessions (N = 30). Test 1: Baseline condition; Test 2: Earplug condition, given approximately a week later; Test 3: Post condition, given the day following Test 2. Error bars represent ±1 SD.

As shown in Figure 1, participants’ state-anxiety scores increased significantly during the earplug condition compared with the baseline and post conditions. A repeated-measures ANOVA revealed a significant main effect of session, F(2,30) = 12.02, *p* = 0.002. Post hoc pairwise comparisons (Bonferroni corrected) indicated significantly higher anxiety during the earplug condition relative to baseline (*p* < 0.001) and post-condition (*p* = 0.005), with no difference between baseline and post (*p* > 0.50). These results suggest that temporary auditory deprivation led to a measurable increase in transient anxiety.

### 3.2. Mood (VAMS)

**Figure 2:**
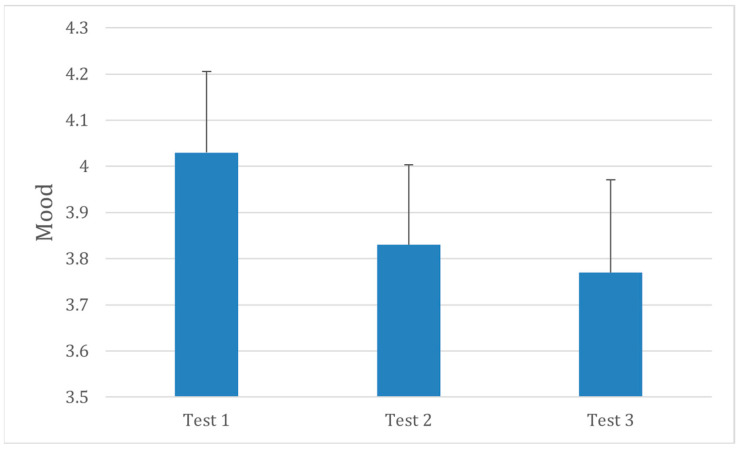
Mean mood levels [VAMS] across three testing sessions. Testing conditions are the same as in Figure 1. Error bars represent ±1 SD.

Mean mood scores decreased slightly during the earplug condition compared with baseline and remained unchanged in the post condition (Figure 2). Although the overall effect did not reach statistical significance, F(2,30) = 1.55, *p* = 0.22, the trend reflected a mild reduction in positive affect while hearing was obstructed. This pattern is consistent with previous reports of transient mood changes following short-term sensory deprivation.

### 3.3. Visual Attention (d2 Test of Attention)

**Figure 3:**
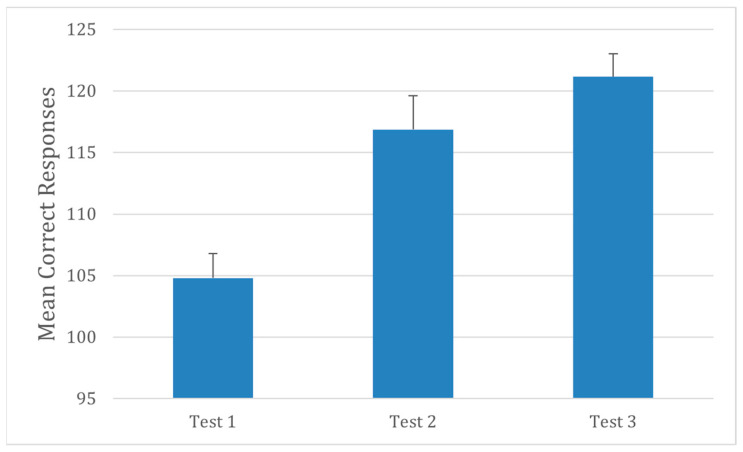
Performance on the d2 Test of Attention across testing sessions (N = 30). Testing conditions are the same as in Figure 1. Error bars represent ±1 SD.

Performance on the d2 Test of Attention improved significantly during the earplug condition (Figure 3). Participants correctly identified more target symbols and achieved higher concentration performance scores compared with baseline and post conditions. A repeated-measures ANOVA confirmed a significant main effect of condition on total correct responses, F(2,30) = 8.42, *p* = 0.001, and on concentration performance, F(2,30) = 5.79, *p* = 0.005. These findings indicate that temporary hearing obstruction enhanced visual selective attention and processing efficiency, possibly due to reduced auditory distraction.

### 3.4. Visual Processing Speed (Coding–Digit Subtest)

**Figure 4:**
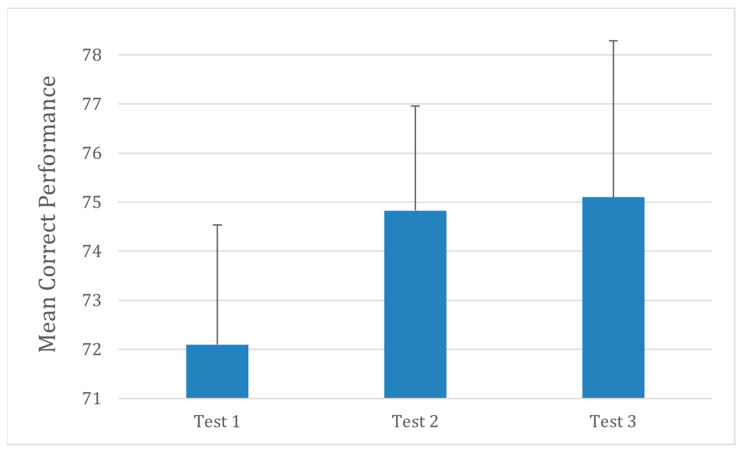
Coding–Digit Subtest performance across testing sessions. Testing conditions are the same as in Figure 1. Error bars represent ±1 SD.

Results of the Coding–Digit Subtest revealed a similar trend (Figure 4). Participants completed significantly more correct symbol–digit pairings during the earplug condition compared with baseline (F(2,30) = 6.33, *p* = 0.004). This improvement persisted slightly in the post condition, suggesting a possible short-term adaptation in visual-motor integration or cognitive resource allocation when auditory input is reduced.

## 4. Discussion

The present study examined the emotional and cognitive consequences of short-term, simulated hearing loss induced by earplugs. The findings revealed that temporary simulated hearing loss produced both negative and adaptive effects: a significant increase in anxiety, a non-significant reduction in mood, and improved performance on tasks of visual attention and processing speed. These results demonstrate that even brief auditory deprivation can influence emotional and cognitive states in measurable ways.

The emotional results align with prior literature reporting associations between hearing loss and increased anxiety or mood disturbances [27,28]. Although most previous studies focused on chronic hearing loss [1], our findings suggest that even short-term auditory deprivation can trigger similar emotional responses. The sensation of reduced sound intensity may evoke uncertainty or discomfort, contributing to heightened anxiety levels. Notably, the persistence of elevated anxiety after earplug removal indicates that emotional recovery may lag behind the restoration of hearing.

In contrast, the improvement in visual attention observed in this study adds a new perspective to the understanding of cross-modal interactions between hearing and vision. Previous studies have shown enhanced visual performance in individuals with long-term deafness, attributed to compensatory neural reorganization [29,30]. Our results suggest that even temporary auditory deprivation may produce short-term attentional benefits, possibly through reduced distraction from irrelevant auditory stimuli or increased motivation to perform visual tasks.

These findings have practical implications for clinical settings. Audiologists and speech-language pathologists should be aware that temporary hearing obstruction—such as that caused by cerumen impaction—may influence patients’ emotional state and attentional functioning during testing. A patient who appears anxious or distracted during hearing assessment may, in fact, be responding to the temporary sensory limitation rather than to the clinical procedure itself.

Can a two-hour-long hearing loss engender noticeable changes in mood and anxiety? The answer is a resounding yes. Sustained emotional effects of even a short negative experience are well documented [31,32]. An argument with a family member in the morning can ruin the rest of your day as can witnessing a violent event on the street. In laboratory experiments, too, brief negative experiences have been shown to produce sustained impact on mood [33,34,35]. In the present study, the mildly negative experience of a noticeable drop in loudness for 2 h induced a (slightly) negative mood that lasted at least for a couple of days

Did the fact that the participants were aware of the temporary nature of manipulation introduce bias? We do not think it did. Many unpleasant exposures in everyday life and certainly in the lab are transient with full knowledge of the participants yet they still induct negative mood. People in the construction industry know in advance that their earplugs are to be placed for short time as do musicians during performance. Advance knowledge does not mitigate reports of discomfort, even anxiety, especially with musicians.

Patients with cerumen-induced hearing problems vary widely in their symptoms. The symptoms are not limited to cerumen per se but extend to many preexisting health issues. It is difficult to isolate the cognitive-emotional effects of cerumen impaction in natural clinical circumstances. This was the reason for the current simulation including solely cerumen-induced impairment with otherwise heathy ears. We also asked our participants to report any existing (or past) hearing problems. We retained only those free of them. Note that the absence of objective hearing tests has no bearing on the validity of the results. Given the within-subject design used, each participant was the participant’s own control across the testing sessions.

Several limitations of this study should be acknowledged. First, the simulation of hearing loss with earplugs may not fully reflect the physiological or emotional experience of patients [not of healthy adults in our case]. Second, patients might not be fully aware of the transient nature of their blockage. Finally, the study employed primarily visual tasks, leaving open the question of how temporary hearing loss affects other sensory or cognitive domains, such as speech perception or motor coordination.

Future research should extend these findings by testing clinical populations with real cerumen impaction and by including neurophysiological measures of attention and emotion. Additionally, it would be valuable to examine whether the observed attentional enhancement persists over longer durations of auditory deprivation.

In conclusion, the present pilot study demonstrates that even short-term, reversible hearing loss can influence both emotional and cognitive functioning. The results highlight the need for clinicians to consider these temporary effects when evaluating patients with ear canal obstruction.

## 5. Conclusions

This first study demonstrated that even short-term simulated hearing loss can influence both emotional and cognitive functioning. Wearing earplugs for two hours led to increased anxiety and reduced mood but also to improved visual attention. These findings suggest that temporary auditory deprivation can have both adverse and adaptive effects. Clinicians and audiologists should consider these short-term changes when evaluating patients with temporary hearing obstruction, such as cerumen impaction.

## Data Availability

The data presented in this study are available on request from the corresponding author due to privacy and ethical restrictions related to participant confidentiality.

## Abbreviations

STPI	State–Trait Personality Inventory
VAMS	Visual Analog Mood Scale
D2	d2 Test of Attention
SD	Standard Deviation
ANOVA	Analysis of Variance
Hz	Hertz
dB	Decibel
